# Non-exclusive exclusion (Commentary on Capello *et al.*)

**DOI:** 10.1111/j.1460-9568.2009.06658.x

**Published:** 2009-02

**Authors:** Diego Rodriguez-Gil, Charles A Greer

**Affiliations:** Departments of Neurosurgery and Neurobiology, Yale University School of MedicineNew Haven, CT 06520, USA

Beginning with their discovery by Buck & Axel (1991), a series of significant advances mark our increased understanding of odor receptors (ORs) and their roles in both odor transduction and axon coalesence (Imai & Sakano, 2008). Somewhat unexpectedly, the family of 1200+ ORs (Zhang & Firestein, 2002) found in the main olfactory epithelium exhibit little homology to those found in the vomeronasal organ where two families, of over 300 vomeronasal receptors (VRs) (Touhara, 2008) mediate pheromone detection. They differ in many respects, including the downstream transduction cascade, but share the elegant property of allelic exclusion, which results in any one sensory neuron expressing one OR or VR from either the maternal or paternal chromosome, but not both. While the significance of allelic exclusion in processing sensory information in the olfactory and vomeronasal systems remains unknown, the mechanisms regulating allelic exclusion are of intense interest.

In this issue, Capello *et al.* (2009) address the question of whether mechanisms of allelic exclusion are similar in both the olfactory and vomeronasal sensory systems. They approached the question using a mouse model in which the odorant receptor M71, normally expressed in the main olfactory epithelium, is now expressed in the vomeronasal organ. This was achieved by using the promoter region of the vomeronasal gene V1RB2 to direct the expression of M71 in vomeronasal sensory neurons in lieu of V1RB2 expression. It was advantageous that M71 has a low percentage of identity with V1RB2 protein, or with any member of the V1R family of vomeronasal receptors.

Previous results showed that axons from new *M71 > V1rb2* neurons target the accessory olfactory bulb where they coalesce to form homogenous glomeruli (Rodriguez *et al.*, 1999). Here, Capello *et al.* (2009) show that these neurons do not express the endogenous V1RB2 receptor or any member of the V1R family of genes. Because members of the odor transduction cascade are important in axon targeting from the main olfactory epithelium (Chesler *et al.*, 2007) and, because the transduction signaling molecules used by main olfactory and vomeronasal sensory neurons are different, the authors then ask whether the expression of M71 also changes the expression of members of the transduction cascade. Vomeronasal sensory neurons express G_alphai2_ and TRPC2, while olfactory sensory neurons express G_alphaolf_, AC3 and CNGA2. Interestingly, *M71 > V1rb2* neurons continue to express markers characteristic of vomeronasal neurons; it appears that their essential identity or phenotype is unchanged other than the expression of M71 in lieu of V1RB2.

Capello *et al.* (2009) show, for the first time, that allelic exclusion in vomeronasal receptor cells can occur through the expression of an exogenous receptor and, that exclusion of expression is not limited to the substituted receptor, but applies to all members of the family – exclusion which is not exclusive. It is tempting to speculate that allelic exclusion may be regulated by universal mechanisms, as suggested by Cedar & Bergman (2008) in considering antigen receptor expression in the immune system. However, the nature of the cascade mediating the one receptor - one sensory neuron rule in the main olfactory epithelium and the vomeronasal system remains elusive. While Capello *et al.* (2009) have shown that M71 effectively excludes expression of V1Rs, and that the axons coalesce appropriately into accessory olfactory bulb glomeruli, the reciprocal replacement is not effective; *V1rb2*>*M71* does not result in viable olfactory sensory neurons whose axons converge in the main olfactory bulb (Feinstein *et al.*, 2004). Contributing to the challenge of understanding these mechanisms in both of these sensory systems is the size of the receptor families and understanding other molecular determinants of differentiation and axon targeting in main olfactory and vomeronasal sensory neurons. Despite many unanswered questions, the current paper from Capello *et al.* (2009) provides a new perspective from which to address the mechanism(s) of allelic exclusion, odor/pheromone transduction, and axon targeting. To paraphrase Shakespeare, would a rose by any other receptor smell as sweet?
